# Skin and Wound Healing: Conventional Dosage versus Nanobased Emulsions Forms

**DOI:** 10.1021/acsomega.5c00455

**Published:** 2025-03-28

**Authors:** João Vitor Vicente-da-Silva, Juliana Oliveira da Silva Lopes Pereira, Flávia Almada do Carmo, Beatriz Ferreira de Carvalho Patricio

**Affiliations:** † PostGraduate Program in Molecular and Cellular Biology, Department of Physiological Sciences − Pharmacology, Biomedical Institute, Federal University of the State of Rio de Janeiro, Rio de Janeiro 20211-040, Brazil; ‡ Pharmaceutical and Technological Innovation Laboratory, Department of Physiological Sciences − Pharmacology, Biomedical Institute, Federal University of the State of Rio de Janeiro, Rio de Janeiro 20211-040, Brazil; § Laboratory of Pharmaceutical Industrial Technology, Department of Drugs and Pharmaceutics, Faculty of Pharmacy, Federal University of Rio de Janeiro, Rio de Janeiro 21941-971, Brazil; ∥ PostGraduate Program in Pharmaceutical Sciences, Faculty of Pharmacy, Federal University of Rio de Janeiro, Rio de Janeiro 21941-971, Brazil

## Abstract

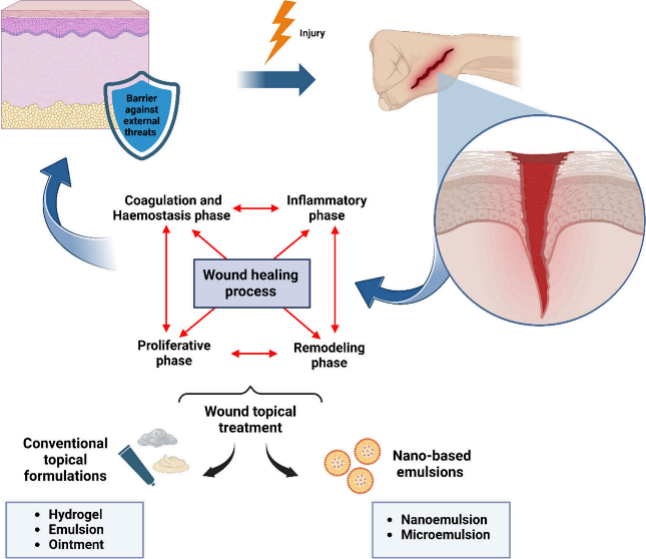

The skin plays a crucial role in the body’s homeostasis through its thermoregulation functions, metabolic activity, and, mainly, its barrier function. Once this system has its homeostasis disturbed, through the promotion of tissue discontinuity, an injury happens and a restoration process starts. Different products can be used to promote, accelerate, or stimulate the healing process, such as hydrogels, emulsions, and ointments (main conventional formulations). Despite the historical use and wide market and consumer acceptance, new systems emerged for wound management with the main challenge to overcome conventional form limitations, in which nanosystems are found, mainly nanobased emulsion forms (nano- and microemulsions, NE and ME). Here, we discuss the skin function and wound healing process, highlighting the cellular and molecular processes, the different wound classifications, and factors that affect physiological healing. We also investigated the recent patents (2012–2023) filed at the United States Patent and Trademark Office, where we found few patents for conventional forms (hydrogels = 5; emulsions = 4; ointments = 6) but a larger number of patents for nanobased emulsions filed in this time (NE = 638; ME = 4,072). Furthermore, we address the use of nanobased emulsions (NE and ME) and their particularities, differences, and application in wound treatment. This work also discusses the challenges, bottlenecks, and regulatory framework for nanosystems, industrial, academic, and government interest in nanotechnology, and future perspectives about this key factor for the nanosystems market and consumer acceptance.

## 1 Introduction

Wounds, especially chronic wounds, are responsible for significant expenditures in healthcare systems worldwide. In the United States of America (USA), it is estimated that at least 2% of the total population may be affected by some type of chronic wound.^1^ A study by Queen and Harding^2^ estimated that the amounts spent on wound management in the three countries with the highest expenditures for this purpose in 2022 (the USA, China, and Japan) were $148.65 billion, $42.78 billion, and $22.91 billion, respectively. Furthermore, the global wound care market is expected to reach $20.33 billion by 2032, growing at a compound annual growth rate of 6.1% from 2024 to 2032.^3^

These data support and highlight that the wound care product market is expanding, as are the annual expenditures by governments and individuals for managing this health issue.^1^ Currently, more than 5,000 wound care products are commercially available, most of which are dressings.^4^ However, there is a strong demand for more effective treatments that reduce hospitalization time, wound management costs, and issues related to patients’ self-esteem and financial burdens. This demand is of great interest to healthcare services, academia, and industry.^5^

Among the technologies that can be employed in developing wound care products are classic formulations (emulsions, hydrogels, and ointments) as well as advanced technological tools, particularly lipid-based systems, such as microemulsions (ME) and nanoemulsions (NE). This work aims to discuss the structure and function of the skin, the wound healing process and its phases, and the factors that affect the normal course of this process. Additionally, it explores both conventional and nanotechnological approaches to wound management and provides a brief survey of patent filings with the United States Patent and Trademark Office (USPTO).

## 2 Skin: Anatomy and Physiology

**Figure 1:**
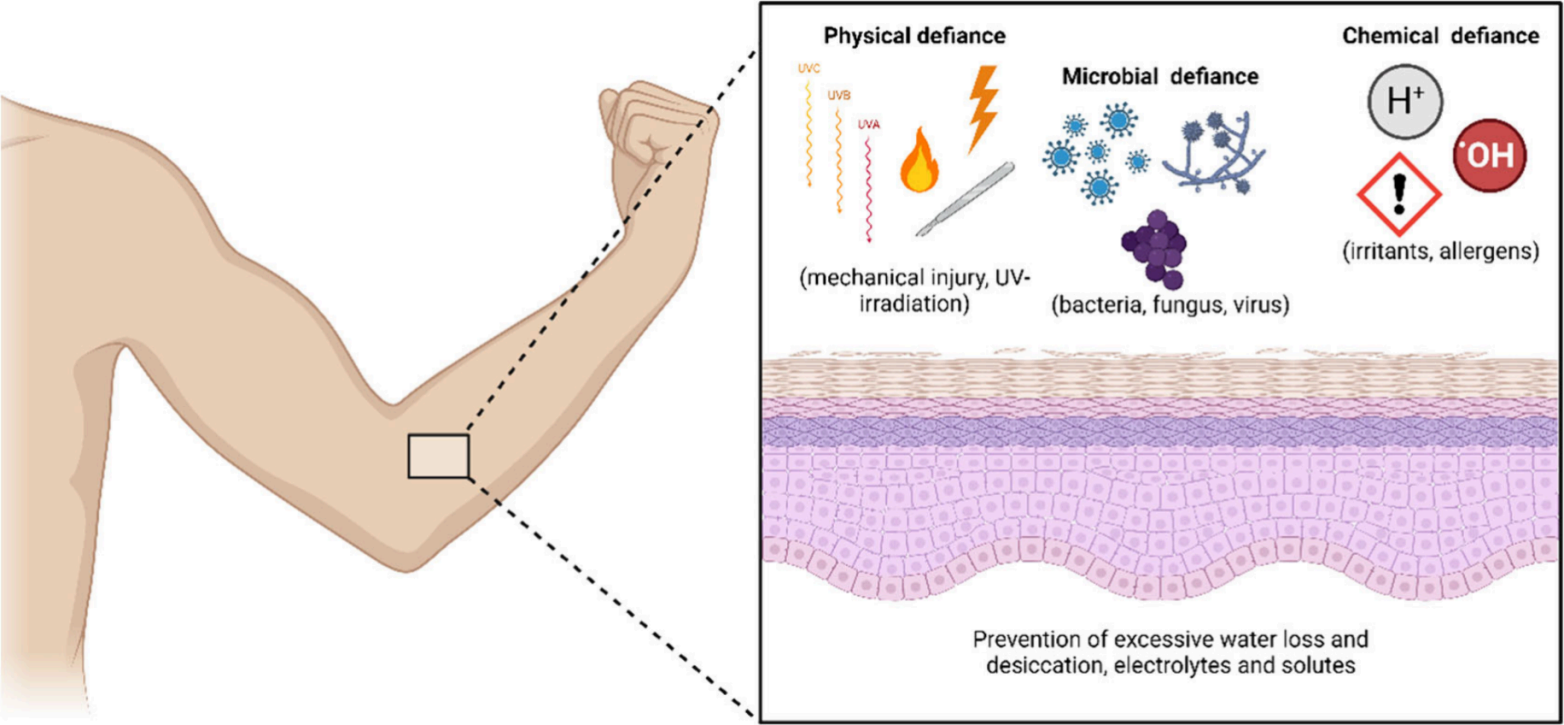
Skin is the border organ of the human body, being at the interface between the internal environment (systems and organs) and the external environment. Among its various relevant biological functions, its barrier action stands out, protecting the organism from external aggressions (physical, microbiological, and chemical), acting as an outside–inside barrier, preventing transepithelial water loss and dehydration, and acting as an inside–outside barrier.

The skin is one of the largest organs of the human body in superficial extension, responsible for around 15–20% of body mass.^6−8^ In an adult weighing 70 kg, for example, the skin weighs around 13 kg.^9^ It has a surface area of about 1.5–2 m^2^ and has the primary function of acting as the body’s outermost protective barrier against external agents, whether physical, chemical, microbiological, or mechanical,^10−14^ and as a protection against the loss of endogenous substances, mainly water from the viable layer of the epidermis and dermis (Figure 1).^15,16^ To assess the importance of the barrier function, the skin retains around 20% of the body’s total water content.^17^ Furthermore, it plays a fundamental role in body thermoregulation, vitamin D production, and sensory perception of the external environment.^7,13^

**Figure 2:**
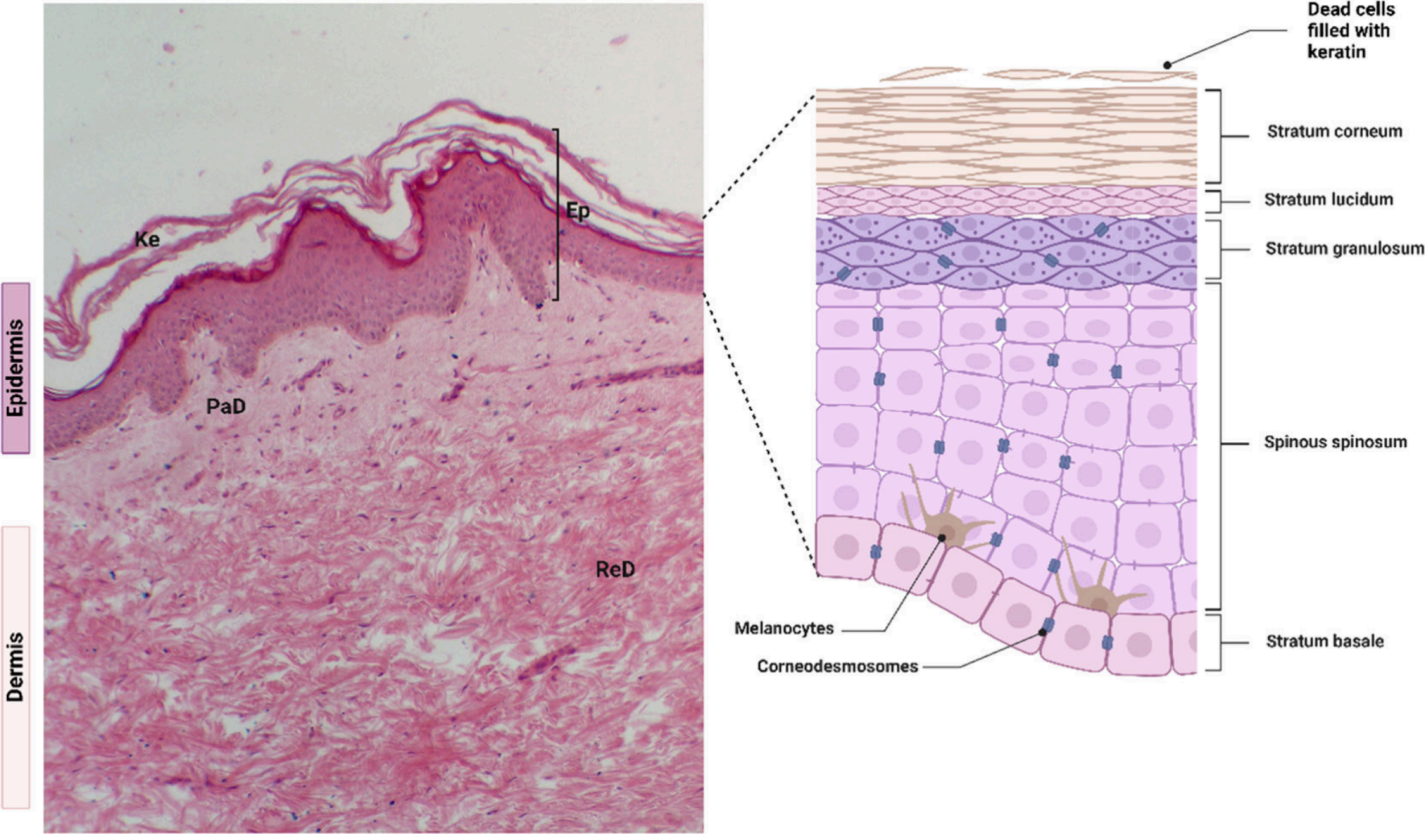
The skin is a stratified tissue, with its basic structures being the epidermis, dermis, hypodermis, or subcutaneous tissue. The epidermis, the outermost layer of the tissue, is mainly responsible for the barrier function and protection against external aggressions. This layer can also be divided into other strata according to the degree of keratinization of the keratinocytes and other intrinsic characteristics, namely, stratum basale, stratum spinosum, stratum granulosum, stratum lucidum, and stratum corneum, with the first three strata known as viable epidermis. The dermis, a layer intimately connected to the epidermis by the dermoepidermal junction, is home to the cutaneous appendages (hair follicles, sweat glands, sebaceous glands, blood and lymphatic vessels, and the neurovascular component of the skin). The dermis is responsible for the support and flexibility of the skin. Ke, keratin; Ep, epidermis (keratinized stratified squamous epithelium); PaD, papillary dermis (loose connective tissue); ReD, reticular dermis (dense irregular connective tissue). The histological section (hematoxylin and eosin-stained sections of human skin) was obtained by the Laboratory of Morphological Sciences, Department of Morphological Sciences of the Biomedical Institute, Federal University of the State of Rio de Janeiro (UNIRIO).

Classically, skin tissue is segregated into two main layers, namely, the epidermis and dermis, with thicknesses of 50–100 μm and 3–5 mm, respectively, with an underlying subcutaneous tissue, also known as hypodermis (Figure 2).^6,10^ Other structures, called cutaneous appendages, such as the hair follicle, sweat and sebaceous glands, and nerve endings, are also constituents of this tissue.^13,18,19^

The epidermis, a keratinized stratified scaly layer and the outermost layer of the skin, has the main functions of providing protection from external aggressors (outside–inside barrier) and the loss of endogenous water and other solutes (inside–outside barrier).^14,20^ It is usually divided into four strata based on the morphology and location of keratinocytes,: from the innermost to the outermost layer, there are the stratum basale, stratum spinosum, stratum granulosum, and stratum corneum.^21^ These divisions result from the continuous process of differentiation of keratinocytes, the primary cell type of the epidermis, and their movement from the innermost strata to the basal layer and to the surface of the tissue.

The dermis is 0.5 to 5 mm thick depending on the anatomical region^9^ and is the layer that supports, nourishes, and connects the epidermis to the subcutaneous tissue, a central structure that provides mechanical resistance to the skin.^22^ Despite having a notable physical barrier capacity and resistance, the skin is also flexible, a capacity conferred by the dermis.^23^ This structure is shaped to resist tears, shear, and localized pressure but simultaneously provide joint movement and local distension/stretching.^24^

This segment is also where hair follicles, sweat glands, sebaceous glands, blood and lymphatic vessels, and the neurovascular component of the skin are housed.^14,20^ All these structures are supported by a polymeric matrix formed by collagen fibers, which provides strength and tenacity; elastin, which provides elasticity and flexibility; and a type of gel composed of glycoproteins and glycosaminoglycans, hygroscopic macromolecular compounds, providing viscosity and hydration.^7,8,25,26^ The primary cell type of the dermis is fibroblasts, responsible for synthesizing and secreting fibers that provide the support characteristics of this skin layer, such as collagen and elastin, and the amorphous components of connective tissue.^27^

Hypodermis/subcutaneous tissue is the deepest layer of the skin. It is designed to act as an energy reserve, thermal insulation, protection for the skin against mechanical stress, and a sliding and anchoring tissue for the underlying structures (dermis, muscle, and bone).^9,28^ Adipocytes mainly form this tissue. A fibrous connective tissue, called septa, delimits the adipocytes, forming lobes. Nerves, blood vessels, lymphatic vessels, fibroblasts, and macrophages are also found in the hypodermis.^25,29^ In non-obese individuals, approximately 80% of all body fat is in the subcutaneous tissue.^9^

## 3 Cutaneous Wound Healing

The discontinuity of the anatomical structure, with consequent alteration of the normal function of a tissue, characterizes wounds. They can be superficial or deep, reaching more external layers or the subcutaneous tissue (hypodermis) and other structures, such as muscles and bones.^30,31^

After the injury (rupture, fissure, or erosion), a multifactorial, complex, and dynamic process begins to reconstruct the tissue and reestablish its previous properties.^29,32^ This process involves several coordinated events such as bleeding, coagulation, initiation of an acute inflammatory response to the initial injury, regeneration, migration, and proliferation of connective tissue and parenchymal cells, as well as synthesis of extracellular matrix proteins, remodeling of new parenchyma and connective tissue, and collagen deposition.^33^

Usually, wounds are classified according to some criteria, which are (i) the type of cause (surgical, traumatic, or ulcerative);^34^ (ii) healing time (acute or chronic wounds);^35^ (iii) microbial content (clean, potentially contaminated, contaminated, or infected/dirty);^36^ (iv) healing (first, second, or third intention);^37,38^ (v) depth (superficial or deep);^39^ and (vi) complexity.^40^

### 3.1 Wounds According to the Type of Cause

Surgical wounds are produced intentionally to manage health issues; therefore, a priori, they cause minimal damage, as they are performed with precision, in controlled and aseptic environments, with the best equipment and recommended approaches, and by qualified professionals.^34,41^ Most of these types of wounds are acute in appearance and follow the healing process adequately, within the expected time and without complications.^42^ They can be performed through an incision, that is, there is no loss of surgically treated tissue and they are generally closed by suture; by excision, when an area of tissue is removed; and by puncture, which is the result of therapeutic-diagnostic procedures, such as catheterization and biopsy.^34^

Traumatic wounds originate from mechanical (e.g., puncturing and cutting), also called laceration,^43^ physical (e.g., cold, heat, or radiation), or chemical damage (e.g., strong acids),^44^ without macroscopic evidence of contamination or active signs of infection.^45^ This subtype of wound is one of the main reasons for seeking emergency care in the USA, with around 6 million lacerations treated annually in emergency rooms.^46^

Finally, there are ulcerative wounds. Ulceration results from tissue necrosis of the skin caused by tissue death due to an ischemic process, often attributed to occlusion of blood vessels.^47^ Ulcers are classified as chronic wounds and comprise the categories of pressure, venous stasis, arterial, neuropathic, and diabetic ulcers.^48,49^

### 3.2 Wounds According to Healing Time

The combination of local and systemic factors, the type and extent of the wound, and underlying diseases determine the direction of the healing process.^35^ Acute wounds tend to repair and resolve the injury, returning to their typical characteristics and functionality at the end of the process and following a healing period of up to 3 weeks.^50^

In the case of chronic wounds, the healing process and its respective phases do not occur in a linear and overlapping manner but rather through a nonlinear and uncoordinated organization.^51,52^ These injuries do not complete all phases of the healing process and remain in a continuous state of nonresolving scar response (mainly in a sustained process of the inflammatory phase).^53,54^ They generally constitute injuries where the scarring process lasts for more than 4–12 weeks.^55−57^ Often, the healing process can take months or years until complete healing, and in many cases, it may not even occur.^58^ A characteristic of this type of injury is that regardless of whether it is superficial or there is a partial or total loss of the entire thickness of the skin, there is a secondary intention healing process (see below).^52^

Chronicity of wounds is typical in individuals who have comorbidities such as diabetes, peripheral vascular diseases, and hemoglobinopathies, as well as in the elderly.^59,60^ Tissue hypoxia, infections, necrosis, exudate, and high levels of inflammatory cytokines are associated with these conditions that polarize the healing process to chronicity.^30^ It is important to highlight that the etiology of chronic wounds in underdeveloped countries may differ slightly from that in developed countries because of conditions such as malnutrition and parasitic diseases such as cutaneous leishmaniasis.^52^

Lack of or inadequate care for these injuries results in amputations and increased mortality.^56^ The most prevalent, incident, and studied types of chronic injuries are pressure ulcers, venous leg ulcers, and diabetic foot ulcers,^52^ which together are responsible for around 70% of all cases of chronic wounds.^61^ Regardless of the type of chronic wound, they share common characteristics, such as excessive levels of pro-inflammatory cytokines, persistent infections, formation of microbial biofilms resistant to treatments, and senescent cells that do not respond to reparative stimuli.^53,62^

It is estimated that at least 1–2% of the world’s population will live with some chronic wounds throughout their lives.^52^ Some authors call the phenomenon of wounds a “silent epidemic”.^63^ In the USA, chronic wounds cost the healthcare system around $50 billion a year.^64^ It is also estimated that in this country, chronic wounds can affect approximately 5–6 million people at any time in their lives.^63^

These types of injuries tend to become what are called complicated wounds, where there is a combination of an infection associated with tissue damage.^50,65^ In addition to the high cost for health systems around the world to manage chronic wounds, there is also the human cost, as the chronically ill individual has a decline in their quality of life resulting from social isolation, anxiety, pain, and a continuous hospital admission cycle.^61,63^

### 3.3 Wounds According to Bacterial Content

As previously mentioned, concerning microbial content, wounds can be classified as clean (Class I), potentially contaminated (Class II), contaminated (Class III), or infected or dirty (Class IV).^36,66^

Class I wounds are performed surgically under aseptic conditions, in which no inflammation is present. They are previously closed (first intention healing), and if they need to be drained, closed wound drain systems must be used.^67^

Class II wounds are related to injuries that access the respiratory, gastrointestinal, genital, or urinary tracts, even in controlled (surgical) situations; due to the degree of invasion of the body and penetration by systems with a wide microbiota, they are considered potentially contaminated.^68^ Surgeries involving the biliary tract, appendix, vagina, and oropharynx are included in this category.^69^

Class III injuries are accidental, fresh, and open wounds.^67^ Furthermore, this group includes wounds resulting from sterile procedures, where for some reason, there is a loss of aseptic conditions during the procedure, such as the leakage of fecal content during the surgical intervention and open-heart massage.^36^ Incisions with acute characteristics and nonpurulent inflammation are also found in this group.^69^ Finally, there are Class IV wounds, which include old traumatic wounds treated improperly or carelessly, with retained or devitalized tissue resulting from a surgical approach, with a previously contaminated site.^36,67^

### 3.4 Wounds According to the Type of Healing

**Figure 3:**
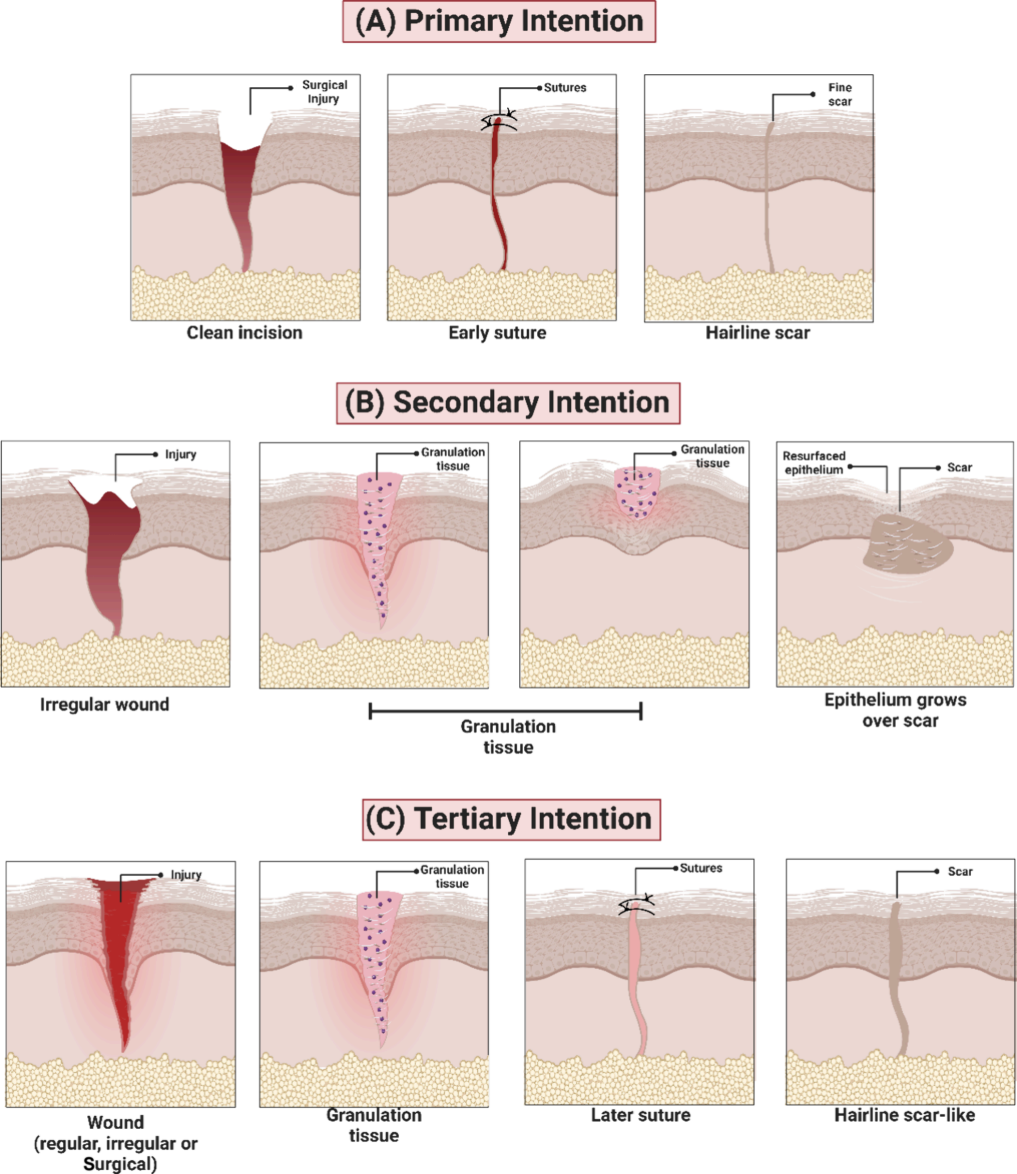
Wound healing processes by (A) primary intention, (B) secondary intention, and (C) tertiary intention. First intention healing is characteristic of surgical processes, where at the end of the procedure, there is a juxtaposition of the edges of the incision made through suturing, for example, favoring a “clean” healing process. Healing by secondary intention is associated with a healing process that occurs organically; that is, it goes through all phases and normal healing processes (coagulation and hemostasis, inflammatory, proliferative, and remodeling phases), culminating in the end in a keloid-looking scar. Finally, healing by third intention consists of a natural wound healing process interrupted after the formation of granulation tissue and surgically corrected to accelerate healing and provide a better aesthetic appearance, for example.

When referring to the type of wound healing, it must be understood that there is a direct relationship with the kind of wound closure, which may be of first, second, or third intention.^55^ In first intention healing, the edges of the wound are juxtaposed, usually through a surgical approach, such as sutures, glues, or adhesive dressings (Figure 3).^58^

Healing by secondary intention is considered a natural process of wound resolution. That is, a surgical approach is not used (Figure 3).^70^ In this scenario, dysfunctions occur in the physiological stages of healing mediated by infections, exacerbated activation of the immune response, or hypoxia that direct healing to the second intention process.^71^ Here, with the loss of tissue, the edges of the wounds are distant, and therefore, the healing process is slower when compared to first intention healing, a characteristic that becomes more striking with the increase in the diameter of the wound.^70,72^ In this case, healing must occur in an open wound from the edges and base of the lesion by depositing new tissue until the wound is completely closed.^73^ Re-epithelialization in this type of healing progresses over the extensively formed granulation tissue, therefore requiring a broad process of tissue remodeling.^71^ Unlike healing by first intention, here is the generation of a striking scar with a characteristic that is not aesthetically pleasing.^67,71^

Finally, there is closure of the third intention wound (Figure 3). In this case, the healing process by secondary intention is intentionally interrupted after the formation of granulation tissue and is corrected surgically, aiming to control a possible infection and provide better functional and aesthetic results to the patient.^67,74^

### 3.5 Wounds According to Depth

In superficial wounds, tissue damage is limited to the most superficial layers, such as the most superficial epidermis or dermis, the papillary layer (or both).^39,75,76^ Deep wounds reach the deepest layers of the dermis (reticular) and can reach adjacent tissues and organs such as adipose tissue, tendons, ligaments, muscles, and bones.^77,78^ The difference in the injury’s depth also helps predict the complexity of the molecular, biochemical, and cellular processes necessary for tissue restoration.^67^ Superficial lesions require regeneration of the epidermis (proliferation and migration of keratinocytes) and the minimal synthesis of new connective tissue. In deep wounds, such as those that require ablation of integumentary tissue, there is a need for the synthesis of new blood vessels, supporting and contractile fibers, and a new epithelium.^61^

### 3.6 Wounds According to Complexity

According to Smaniotto and co-workers,^79^ simple wounds are defined as those that resolve spontaneously and usually follow the four phases of the physiological healing process (see below).^79^ Furthermore, the Doctors Without Borders wound treatment guideline^80^ indicates that simple wounds are limited to reaching the subcutaneous tissue without losing or damaging the underlying structures.

Complex wounds can be acute or chronic, with complicated resolution.^81^ Ferreira and co-workers^82^ defined criteria to classify the complexity of wounds: (i) extensive tissue loss, (ii) presence of important infections, (iii) compromised tissue viability (presence of ischemia and/or necrosis), and (iv) underlying diseases that may disrupt the normal healing process, such as diabetes.^81−83^ Wounds in diabetic patients, pressure ulcers, chronic venous ulcer wounds, extensive necrotic events caused by infection, such as Fournier’s syndrome, wounds related to vasculitis and immunosuppressive therapies that did not heal in previous treatments, and burns can be classified as complex wounds.^82^

### 3.7 Wound Healing Process

The wound healing process is didactically divided into phases/steps for better understanding, despite all processes being interdependent and temporally overlapping, namely, the (i) hemostasis phase, (ii) inflammatory phase, (iii) proliferative phase, and (iv) remodeling phase.^30,50,84,85^

#### 3.7.1 Coagulation and Hemostasis Phase

Following the injury, hemostasis processes begin. Initially there is a reflex response of vasoconstriction of the smooth muscles surrounding the blood vessels, intending to prevent bleeding and protect the vascular system and vital organs,^32,85,86^ mediated by sympathetic discharge and thromboxane A2, for example. This reflex contraction is essential during a short period following the injury (about a few minutes), as there is immediate vasodilation due to hypoxia and established local acidosis, with a return to bleeding.^31^ Therefore, this hemostatic activity of the capillaries would be insufficient without the coagulation component.^64^

**Figure 4:**
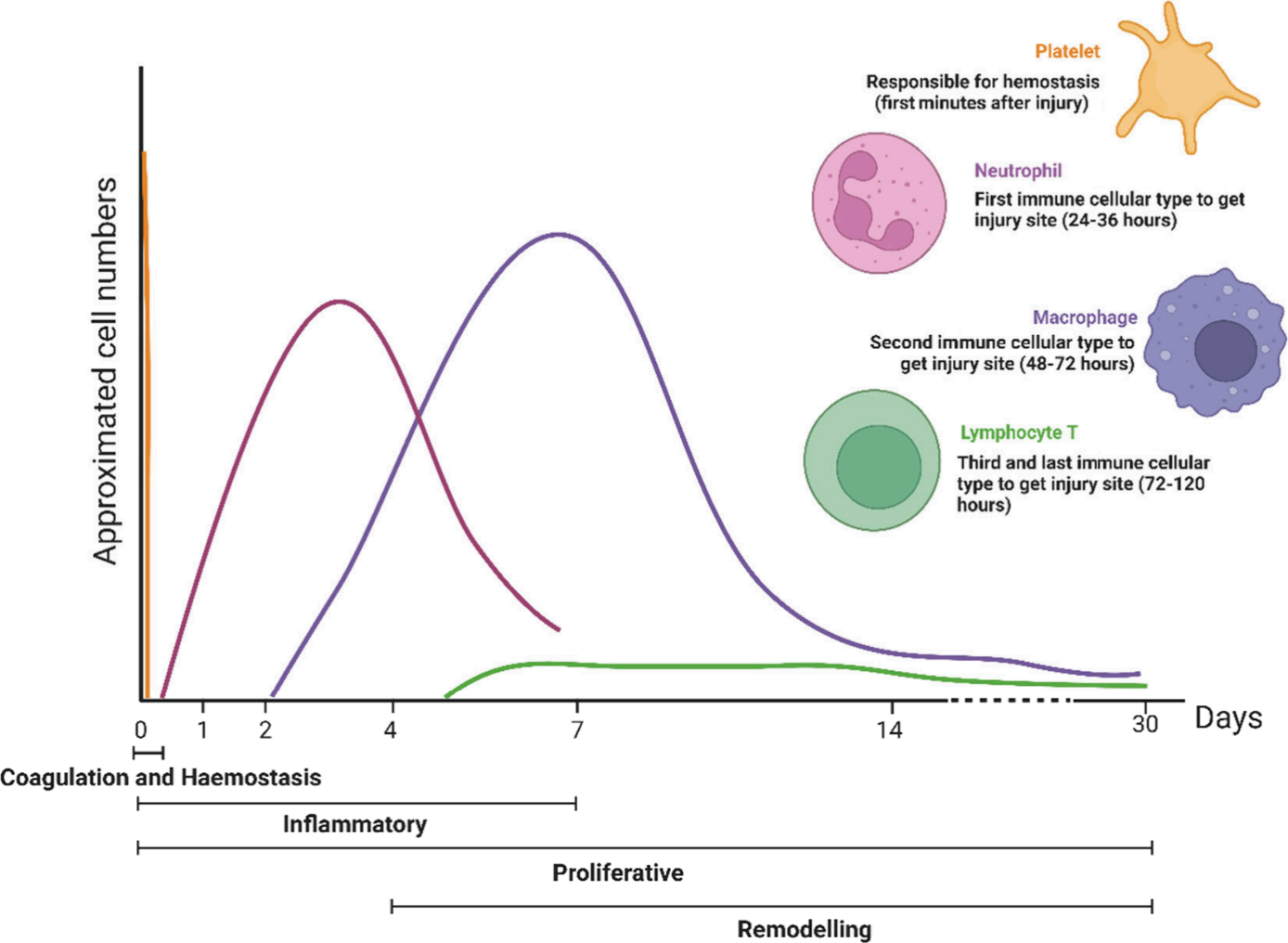
Main blood and immune cell types that participate in wound healing in the different phases of the healing process (coagulation and hemostasis, inflammatory, proliferative, and remodeling phases). Initially, platelets form a platelet plug to prevent too much blood loss. This process occurs on the scale of minutes. Neutrophils, the most abundant leukocyte in the blood, are the first cell type of the immune system to migrate to the injury site and begin the inflammatory phase of healing. Macrophages migrate secondarily and are critical cells in the progression of healing to the proliferative phase. Finally, T lymphocytes migrate at the end of the inflammatory phase and remain at the injury site until the healing process ends.

Then, with blood extravasation into the extracellular space, blood components, such as platelets, encounter extracellular matrix proteins (e.g., fibronectin, collagen, and von Willebrand factor),^57^ and they aggregate, forming a platelet plug, a process that is called primary hemostasis (Figure 4).^84^ Secondary hemostasis consists of the activation of the coagulation cascade through the extrinsic and intrinsic pathways, forming an insoluble fibrin network, configuring a thrombus that stops bleeding, in addition to being a first line of defense against the invasion of pathogenic microorganisms.^60,87^ The formed thrombus is also important in the following phases of the coagulation process, as it acts as an adhesion matrix for the infiltration of other cell types.^64,88^

Platelets trapped in the clot have granules containing platelet-derived growth factor (PDGF), transforming growth factor-β (TGF-β), epidermal growth factor (EGF), platelet-activating factor (PAF), fibroblast-2 growth, adenosine diphosphate, insulin-like growth factors, and chemoattractant molecules for cells necessary for the healing process, such as neutrophils, macrophages, endothelial cells, and fibroblasts.^14,33,50,54,61,89^

#### 3.7.2 Inflammatory Phase

After these initial phases, the inflammatory phase begins, where the humoral and cellular inflammatory component establishes an immunological barrier against possible invading pathogens,^64^ a response initiated by chemoattractant cytokines secreted by platelets and adjacent cells to the site of injury but also by signs of injury, such as molecular patterns associated with damage and molecular patterns associated with pathogens.^57^ That is divided into two stages: (i) the initial inflammatory phase and (ii) the late inflammatory phase.^90^ The first phase aims to initiate the complement system and molecular events that culminate in the infiltration of neutrophils, which have the primary role of initially phagocytizing bacteria, foreign particles, and damaged tissues at the site of the injury.^32,60,91^ Neutrophils act through the phagocytosis of cellular debris and pathogens, the release of reactive oxygen species into the extracellular environment, antimicrobial peptides, eicosanoids, and enzymes with proteolytic activity.^57^ Furthermore, these cells secrete a DNA network containing histones, proteases, and other proteins capable of trapping and killing pathogens, called the neutrophilic extracellular trap.^52^

This cell type is attracted to the injury site within 24–36 h (Figure 4)^14,89^ through several chemokines, such as prostaglandins, tumor necrosis factor-α (TNF-α), PAF, TGF-β, C3a and C5a, and bacterial endotoxins, such as lipopolysaccharide.^57,65,87^ In addition to the secretion of these substances by the platelets and endothelial cells adjacent to the site of injury, the mast cell also plays a prominent role in the chemotaxis of neutrophils, mainly through the secretion of histamine.^64^

Increased expression of adhesion molecules on the endothelial surface (P-selectin, E-selectin, vascular cell adhesion molecule-1, and integrins) and their respective ligands on neutrophils allows rolling and diapedesis to the inflamed site, where neutrophils will perform their functions.^29,84^ After controlling bacterial contamination, for example, it is necessary to eliminate this immune cell to continue the healing phases. Therefore, neutrophils undergo apoptosis, phagocytosed by macrophages, and emigration or efferocytosis, for example.^92^

In the late inflammatory phase, the prominent cell type is macrophages, which appear approximately 48–72 h after the injury and continue the process of phagocytosis of cell debris at the injury site (Figure 4).^33^ Like neutrophils, macrophages migrate from the bloodstream through diapedesis.^32,93^

The presence of these cells in the inflammatory site is important, as they are regulatory cells of the type of immune response, carrying out the transition between inflammation and the proliferative phase, and are reservoirs of growth factors such as TGF-β, transforming growth factor-α (TGF-α), and vascular endothelial growth factor (VEFF) activating keratinocytes, fibroblasts, and endothelial cells.^35,50^ Furthermore, macrophages link innate and adaptive immunity by presenting antigens to T lymphocytes locally in the wound bed. Finally, the last inflammatory cells to migrate to the injured tissue 72 h after the injury are lymphocytes (Figure 4).^93,94^

#### 3.7.3 Proliferative Phase: Angiogenesis, Granulation Tissue, and Epithelialization

The proliferative phase begins 3 days after wound induction (72 h) and lasts 2 weeks.^85,87,92^ It is marked by fibroblast migration, a new extracellular matrix deposition, angiogenesis, re-epithelialization, and repair of peripheral nerves.^29^ At a macroscopic level, it is possible to identify this phase by the abundant formation of granulation tissue.^33^ Granulation tissue formation begins within the first 4 days after the injury.^88^ The tissue has a pink, soft, and grainy appearance.^95^ This characteristic can be observed below the formed wound crust. It comprises fibroblasts, newly formed blood vessels, collagen fibers, mainly type III, and elastin.^71^ This tissue, primarily the developed extracellular protein network, provides a substrate for migrating fibroblasts and keratinocytes from the edge to the center of the injury.^96^

During the initial period of the injury, the surrounding fibroblasts are stimulated to proliferate and deposit collagen, a process called fibroplasia.^97^ After this time, they migrate to the wound attracted by substances such as fibroblast growth factor-2 (FGF-2), PDGF, and TGF-β secreted during the inflammatory phase and proliferate profusely, secreting products from the extracellular matrix, such as hyaluronic acid, fibronectin, proteoglycans, and type I and III collagens.^87^

In normal skin, type I collagen is responsible for around 80% of the content of this protein type, while type III is responsible for 15%. However, in the healing process, this proportion is reversed in the skin, and type III becomes the predominant collagen.^60,88^ As the healing phases progress, this parameter tends to normalize.^50,89^ Initially, hyaluronic acid helps the tissue resist compression, and fibronectin acts as a substrate for the fixation of the cell itself.^32^

After 1 week, there is already an abundant extracellular matrix composed mainly of collagen, supporting cell migration and strength and elasticity to the tissue, for example. At this moment, there is a contraction of the tissue, promoted by the change in the phenotype of the fibroblasts on the wound margins to myofibroblasts, cells with contractile capacity.^54^ This contraction is vital in the healing process, as it brings the edges of the injury closer together.^64^ It can happen at 0.75 mm daily.^60^

During the proliferative phase, it is also important to establish new blood vessels, a process known as angiogenesis, for the exchange of gases and supply of nutrients to new cellular components in development.^86^ Conditions such as ischemia in the injured tissue, increased lactate concentration, and decreased pH and O_2_ saturation contribute to neovascularization.^35,60,61^ During the previous phases, several angiogenic factors are secreted by macrophages, keratinocytes, endothelial cells, and fibroblasts, such as TGF-β, TGF-α, angiogenin, and PDGF, for example.^50,65^

At this stage, there is still progress in the re-epithelialization of the wound, starting in the first hours of injury (16–24 h), due to the production of the necessary cellular support (extracellular matrix).^88^ This process always begins at the edges of the lesion, a phenomenon called “free neighborhood effects”, ending when the re-epithelializing edges are in the center of the lesion.^92^ The objective is to restructure the biological functions of the epidermis that were lost with the injury, such as mechanical protection, temperature regulation, and water barrier.^98^

At the end of this phase, the wound bed is filled with granulation tissue, and angiogenesis re-establishes blood flow and promotes the progressive regeneration of the lymphatic network. Gradually, the granulation tissue strengthens with more collagen fibers, and as a result, the injured area takes on the appearance of a scar due to the accumulation of fibrous masses.^57,99^

#### 3.7.4 Remodeling Phase

The final healing phase characterizes it and is responsible for the new epithelium’s maturation and scar tissue formation.^88^ This phase can generally last around 1 to 2 years^35^ and is highly regulated by a balance between synthesis and degradation of the protein and cellular components of the newly formed tissue to organize the structure to achieve the characteristic closest to that of the preinjury tissue. A skin tissue scar can restore around 70–80% of the skin’s original resistance.^50,87,89^ The ability to achieve maximum resistance close to the tissue of origin depends on the size, depth, location, and type of wound (traumatic or surgical), nutritional status, the severity of the wound, treatment, and the individual’s general health status.^86,88^

At the end of this phase, the regeneration of skin appendages, such as hair follicles and glands, is limited, and scars become paler due to insufficient regeneration of melanocytes and low vascularization of the region.^100^

### 3.8 Factors Affecting Wound Healing

The healing process is susceptible to several factors that can make it challenging to resolve the injury.^101^ The imbalance of cellular and molecular interactions necessary for wound healing can result in errors in the process, leading to delays and possible tissue compromise.^102^

**Figure 5:**
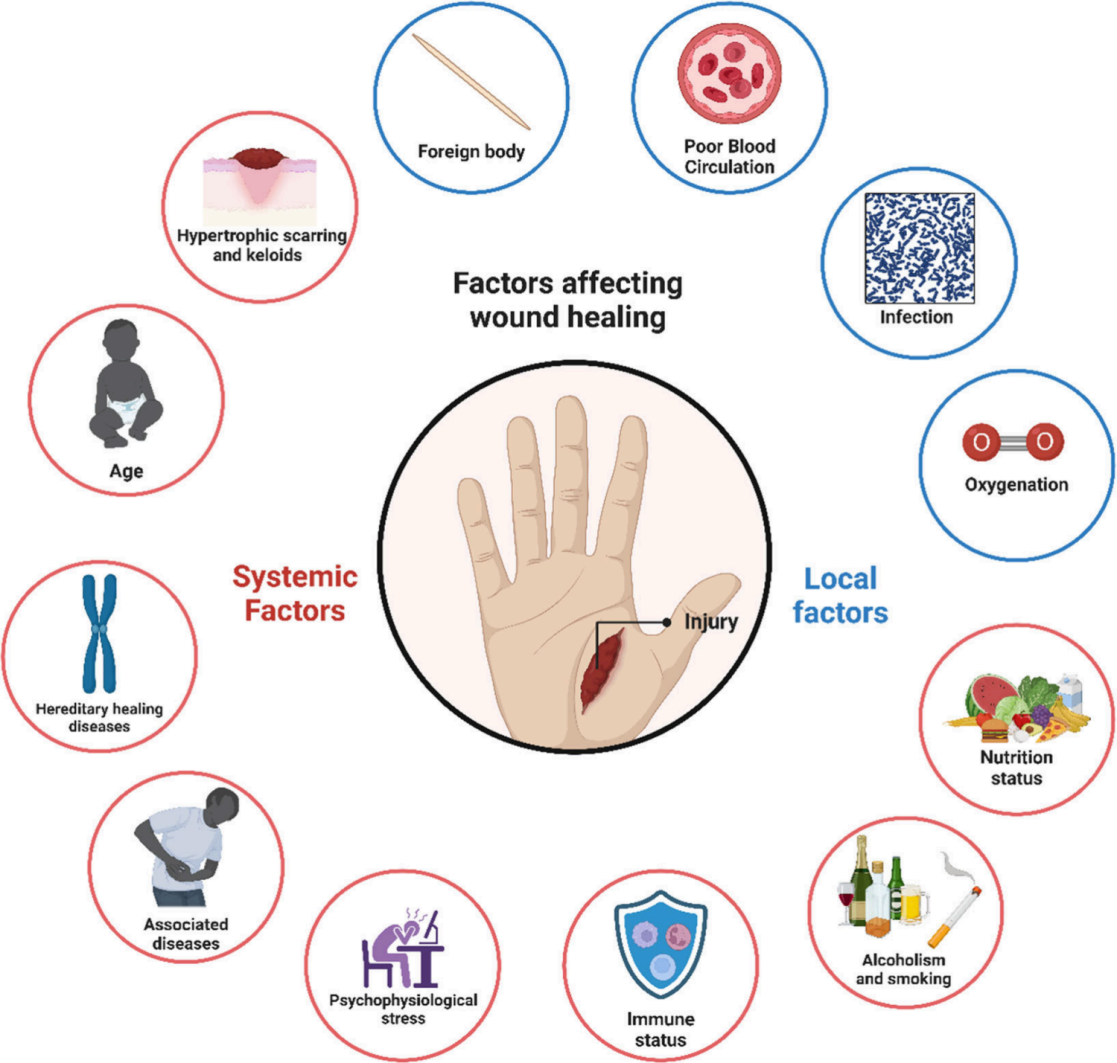
The physiological healing process can be affected by local or systemic factors that make the healing of an injury difficult. The presence of foreign bodies, infection, and low oxygenation are examples of local factors. At the same time, age and gender, sexual hormones, stress, associated diseases such as diabetes and obesity, nutritional status, immune system status, and lifestyle habits, such as a sedentary lifestyle, alcoholism, and smoking are categorized as systemic factors, as they affect the individual’s overall health, as well as the ability of healing mechanisms to resolve the injury.

The factors influencing repair can be categorized as local or systemic (Figure 5), and many can be closely related.^103,104^ Local factors are associated with the site of the injury itself, while systemic factors affect the individual’s health or their ability to heal the wound.^105^ Local factors include the presence of foreign bodies, hypoxia, tissue ischemia, and the presence of biofilm or bacterial infection, for example.

In hypoxia, the microenvironment at the site of injury signals the release of mediators that coordinate the mechanisms of angiogenesis, re-epithelialization, and synthesis of growth factors and cytokines; however, hypoxia leads to the synthesis of reactive oxygen species (ROS) and pro-inflammatory cytokines that can harm the healing process.^102,104^ In infections, leukocyte migration and phagocytic activity lead to the release of endotoxins by bacteria, resulting in necrosis and local inflammation due to an increase in pro-inflammatory cytokines, greater metalloproteinase activity, and a decreased release of growth factors.^91,105^ Chronic inflammation impairs the healing process, affecting re-epithelialization and delaying wound retraction and tissue remodeling.

Concerning systemic factors, comorbidities stand out, such as diabetes, belonging to the extremes of the age group, malnutrition, and genetic factors,^103^ with age being one of the main risk factors related to impaired healing due to metabolic and systemic changes resulting from aging, such as a delay in the migration of leukocytes to the area of injury, a decrease in the phagocytic activity of macrophages, and a decrease in the thickness of the epidermal layer and collagen remodeling.^102^

Nutrition is also of great relevance since the absence of substrates necessary for the regenerative process, such as vitamins, fatty acids, minerals, and proteins, impairs healing by prolonging inflammation, decreasing phagocytosis, collagen synthesis, and the mechanical resistance of the skin, in addition to delaying neovascularization.^101,106^ Even stress can affect wound healing through the increased release of molecules such as epinephrine and norepinephrine, cortisol, and glucocorticoid hormones, which promote a decrease in the release of cytokines and the leukocyte immune response.^102^

Among comorbidities, diabetes commonly leads to impaired healing of acute wounds.^105^ Hyperglycemia generates high levels of metalloproteinases, which inhibit repair processes, and of the so-called advanced glycation products, which in several in vivo tests have been shown to accumulate in skin collagen, altering its structure, and lead to oxidative stress through the production of ROS, generating apoptosis of endothelial cells and fibroblasts.^105^

Furthermore, alcoholism and smoking are also considered systemic factors that interfere with healing. Acute exposure to alcohol results in suppression of the release of pro-inflammatory cytokines, decreased neutrophil recruitment and phagocytic function, reduced angiogenesis, and collagen production, in addition to increasing susceptibility to infections.^105,107^ Cigarette compounds such as tobacco, nicotine, carbon monoxide, and hydrogen cyanide generate hypoxia, reduce the migration of fibroblasts, impair collagen synthesis, epithelial regeneration, and angiogenesis, and increase platelet aggregation, which can lead to thrombosis and embolism.^102,107^

## 4 Classical Topical Formulations Used for Wound Treatment

The market provides evidence of the preference and acceptability of products for topical administration for these formulations. In 2018, the topical benefits market profited from around $11.21 billion,^108^ and each year, 60.5 billion units of discussed medicines are sold worldwide.^109^ In 2022, the global market for wound treatment products reached the mark of $21.4 billion, with a projected growth of 4.15% between 2023 and 2030.^110^ In therapy, some alternatives can be used to treat wounds, emphasizing nonsurgical approaches. They aim to debride the wound bed, maintain the moisture balance at the site, and control inflammation, infection, re-epithelialization, and wound contraction.^5,102^ Among these approaches are some so-called conventional or classic pharmaceutical forms, such as gels, emulsions (creams and lotions), ointments, pastes, foams, and sprays, among others.^5,111^ The literature indicates that topical wound management products, such as those in Table 1, must have ideal dressing characteristics.

**Table 1:** Ideal Characteristics of Supplies for Wound Treatment^a^

high moisture vapor permeability, i.e., allowing a wound to “breathe”
lack of adhesion to the core of scar tissue
high absorption capacity
set up a barrier against external contaminants (chemical or microbiological)
capable of sterilization
possess good adhesion to wound edges
hypoallergenic
sensorially pleasing
cost-effective

a Adapted from Sarabahi.^112^

In the following sections, we will highlight hydrogels,^113^ emulsions,^114^ and ointments.^115^ These pharmaceutical systems are highlighted because of their historical use in pharmaceutical and cosmetic products for topical application to treat a wide range of dermatological conditions, mainly wound healing. Hydrogels have a vast literature about their chemical, usage, and biological and environmental safety since the first mention in scientific reports dating from 1960.^116,117^ Emulsions date from prehistory, and ancient Chinese, Egyptians, Indians, and even Mayans used “emulsion technology” in their routine in food and hygiene.^118^ Likewise, ointments were used for skin care needs and as medicine by Egyptians and Babylonians in 3,000 BCE.^119^ At the same time, these three systems represent the primary pharmaceutical forms for topical drug products on the market.^119^

### 4.1 Hydrogels

Hydrogels are defined as a polymeric network with a three-dimensional configuration with the ability to trap and interact with a large amount of water or biological fluids (>90%).^120,121^ These systems are widely used on different fronts, such as in the food industry, tissue engineering, and pharmaceuticals, to deliver active ingredients.^122^ Hydrogels can be classified according to several characteristics. However, the main one refers to the nature of the polymer as natural (e.g., collagen, hyaluronic acid, cellulose, gelatin) or synthetic (e.g., PEG derivatives, cellulose derivatives, poly(vinyl alcohol)).^120,123^

In general, classic hydrogels have several advantages for topical application, such as providing hydration, a pleasant appearance to the touch, protecting the administration site against the external environment, biocompatibility, biodegradability, and being nontoxic.^124^

Furthermore, hydrogels promote a controlled release and provide desirable characteristics for topical application, such as increased viscosity, adhesiveness, and spreadability, in addition to being easily washable, thus enabling greater adherence to treatment by the user.^125−127^ Also noteworthy is the protection provided to the active substance through the prevention of enzymatic degradation, oxidation, and hydrolysis, for example, increasing the useful life of the product.^121^

Thinking about an application to wounds, hydrogels, due to their high amount of water, keep the wound moist, favoring cell growth, proliferation, and migration, in addition to collagen synthesis.^120^ Added to this, they can reduce the temperature of the injury, while their high amount of water helps to soften and hydrate the affected area, an essential element in the care of dry wounds, such as burns. Additionally, they absorb the exudate and move it away from the lesion region, resulting in a notable reduction in the risk of secondary infections.^120,128^

However, as for limitations, as hydrogels are essentially aqueous-based, they are not viable to incorporate lipophilic substances into their formulation.^129,130^ Furthermore, hydrogels are difficult to sterilize and are prone to water loss into the environment, leading to the breakdown of the formulation.^120^

Table 2 presents the patents filed between 2012 and 2023 with the USPTO related to hydrogels for wound management.

**Table 2:** Patents Filed on Hydrogels to Treat Wounds (2012–2023)

hydrogel				
patent title	publication information	date of publication	inventor	reference
metabolized conditioned growth medium and methods of use	US 20120121522 A1	2012-05-17	Gruber et al.	(131)
formulation for topical wound treatment	US 20160324971 A1	2016-11-10	Kilic; Cicek	(132)
topical compositions and methods for treating wounds	US 20170136086 A1	2017-05-18	Chacon; Saenz	(133)
topical erythropoietin formulations and methods for improving wound healing with and cosmetic use of the formulations	US 20180243373 A1	2018-08-30	Hamed	(134)
pharmaceutical composition for topical wound treatment	US 20210267976 A1	2021-09-02	Ramos Vernieri et al.	(135)

### 4.2 Emulsions

Emulsions are classified as colloidal dispersions. Macroscopically, they are characterized as a homogeneous system, but at the microscopic level, they are heterogeneous.^136^ This characteristic is because an emulsion is a mixture of two or more immiscible liquids, often oil and water. As is generally known, oil and water do not mix spontaneously. Thus, a third component, a surfactant, is used, which promotes the mixing of liquids by reducing the interfacial tension between them.^137^ For this reason, emulsions are thermodynamically unstable systems or, according to some authors, a metastable system, that is, they “should not exist” but can be observed, even if for a short time.^138^ This implies two points: (i) there is a need to supply energy for the formation of an emulsion; (ii) the formulation will invariably break, and phase separation will occur.^139^

The surfactant promotes the entrapment of one of the liquids within droplets, called the internal, dispersed, or discontinuous phase. In contrast, the other phase is called the external, continuous, or dispersing phase. Therefore, the emulsion stays stable for a longer period.^140^ The oil:water ratio and the surfactant used can predict the relationship between which liquid (oil or water) will be one or the other phase.

Surfactants with hydrophilic–lipophilic balance (HLB) values between 3 and 6 tend to form water emulsions in oil, while HLB values between 8 and 18 form oil-in-water emulsified systems. In addition to these two main types of emulsions, a more complex one can be created with multiple phases, such as water-oil-water or oil-water-oil.^141^ Pharmaceutical or cosmetic emulsions vary according to their consistency, from lotions (low viscosity) to creams (high viscosity).^136^

Like any other system, emulsions have their advantages and limitations. Emulsions can carry substances of both a hydrophilic and a lipophilic nature, increasing the absorption of substances. They can mask the odor or flavor of active ingredients if they remain in the dispersed phase, improving the palatability of the final formulation.^137^ However, this system’s main disadvantage is its thermodynamic instability, which ultimately results in phase separation and limits the product’s useful life.^136^

Regarding the application of emulsions to wounds, their emollient properties can be highlighted, providing the wound site with hydration and nutrition. They are quickly absorbed by the skin, and their intrinsic amphiphilic characteristic provides the delivery in a single system of hydro- and lipophilic substances.^142^

Table 3 presents the patents filed between 2012 and 2023 with the USPTO related to emulsions for wound management.

**Table 3:** Patents Filed on Emulsions to Treat Wounds (2012–2023)

emulsion				
patent title	publication information	date of publication	inventor	reference
metabolized conditioned growth medium and methods of use	US 20120121522 A1	2012-05-17	Gruber et al.	(131)
skin cream	US 20120225029 A1	2012-09-06	Al-Qahtani	(143)
composition for use in reducing scab formation and promoting healing	US 20160081968 A1	2016-03-24	Svensson; Linsefors	(144)
topical compositions and methods for treating wounds	US 20170136086 A1	2017-05-18	Chacon; Saenz	(133)

### 4.3 Ointments

The third and final type of system presented here is ointments. Ointments are homogeneous, high-viscosity systems, commonly with a greasy/oily sensory appearance (usually 80% oil), and are intended for topical application.^145^ Like the pharmaceutical forms mentioned above, ointments may or may not contain active ingredients. In the latter case, they are used due to their protective, emollient, or lubricating properties.^146^

According to Asija and co-workers,^146^ ointment bases are classified by the United States Pharmacopeia into hydrophobic, absorption, water-removable, and water-soluble bases. The choice of base is based on some criteria, namely, (i) the desired speed for the release of the active substance from the base, (ii) whether topical or transdermal action is aimed at, (iii) whether occlusion of the skin is desirable, (iv) breaking the stability of the active ingredient in the ointment, (v) the interference of the active ingredient in the consistency of the system, (vi) whether the objective is to easily wash off the base or not, and finally, (vii) the characteristics of the surface on which the system will be applied.

Although its application is an excellent choice for the management of some conditions, such as the treatment of dry skin,^145^ psoriasis,^147^ and atopic dermatitis,^148^ regarding wound treatment, the adjustment for choosing the ideal base is finer, as the high lipid characteristic of the formulation gives a rancid and unpleasant sensation to the application.

Table 4 presents the patents filed between 2012 and 2023 with the USPTO related to ointments for wound management.

**Table 4:** Patents Filed on Ointments Aimed at Treating Wounds (2012–2023)

ointment				
patent title	publication information	date of publication	inventor	reference
metabolized conditioned growth medium and methods of use	US 20120121522 A1	2012-05-17	Gruber et al.	(131)
topical wound treatment method and composition	US-20150024053 A1	2015-01-22	Harrison; Hodges	(149)
composition for use in reducing scab formation and promoting healing	US 20160081968 A1	2016-03-24	Svensson; Linsefors	(144)
topical compositions and methods for treating wounds	US 20170136086 A1	2017-05-18	Chacon; Saenz	(133)
compositions and methods for keloidless healing	US 20190374556 A1	2019-12-12	Takabe; Aoki	(150)
wound treatments and compositions	US 20220047590 A1	2022-02-17	Ray	(151)

It is possible to observe that a few patents have been published at USPTO related to classical formulations in the last 10 years. There can be some explanations for this data. Although hydrogels, emulsions, and ointments are excellent, efficient, and cheap alternatives to carry drugs to the wound bed, their intrinsic limitations remain.

In this scenario, new strategies to develop carrier drugs emerge as an interesting mechanism to treat old health threats, e.g., nanotechnology. With the expansion of nanotechnology in various fields of science, the use of nanotechnology tools by the pharmaceutical industry has expanded, so-called nanosystems.

Nanodimensional materials can give new physical-chemical properties to substances and modulate their interaction with biological systems.^152^ As a result of these new properties, it can be to emphasize the increase of skin’s permeation, to connect to the intimate contact with the epidermis, the particles’ nanometric size, and the contact time with the skin; nanosized materials can protect drugs against physical and chemical instabilities, provide a drug-controlled release, and reduce toxicity and side effects.^6,153^

Herein we highlight nanoscale emulsions (NE and ME). They have been widely investigated as delivery systems of pharmaceuticals for the skin.^153^

## 5 Nanoemulsions and Microemulsions for Wound Treatment

The benefits of using NE and ME for wound management are associated with the physicochemical characteristics of the nanosystems, such as increased solubility of the active ingredient, increased bioavailability, easy preparation, and controlled release of active ingredients, among others.^154^ In addition, these nanosystems are composed of biocompatible and biodegradable components, reducing potential adverse effects.^124^

Furthermore, some studies have demonstrated the ability of NE to modulate inflammatory activity,^155^ increase the proliferation and cell migration of keratinocytes,^156,157^ and have antibacterial activity,^158^ in addition to promoting wound healing in an animal model.^155^ Regarding ME, some studies point to antimicrobial and anti-inflammatory activities and its in vitro and in vivo healing potential.^159−161^

Arpa and co-workers^161^ evaluated the in vitro wound healing potential of an ME containing benzocaine and fusidic acid in the L929 fibroblast cell line over a 24 h period. Benzocaine is a local anesthetic that can be used topically for pain relief in wounds, while fusidic acid is an antimicrobial agent with demonstrated efficacy in the wound healing process.^163^ In vitro assays serve as a preliminary screening method and offer several advantages, such as result reproducibility, rapid data generation, and a preliminary assessment for subsequent in vivo studies.^164^ The authors observed that the ME exhibited a higher percentage of wound contraction compared to the control group (91.46 ± 4.50 > 78.07 ± 3.21%). Lôbo de Souza and co-workers^165^ developed NE containing vegetable oils from different species of wild passion fruit, namely *Passiflora alata* (PA-NE), *Passiflora cincinnata* (PC-NE), *Passiflora setacea* (PS-NE), and *Passiflora tenuifila* (PT-NE). They conducted a cell proliferation assay using the HaCat keratinocyte cell line at concentrations ranging from 3.125 to 100 μg/mL. It is well-known that the fatty acids present in passion fruit seed oils, particularly oleic and linoleic acids, exhibit wound healing activity and promote cell proliferation.^165−167^ All oils and their respective NE demonstrated proliferative effects with the NE showing a delayed onset of action, which is expected for these nanocarrier systems as drug delivery platforms.

Other studies demonstrate the effect of nano- and microemulsions in animal models. Ahmad and co-workers^168^ developed an NE containing curcumin (Cur-NE) and evaluated its healing (over 24 h) and anti-inflammatory effects in albino rats, comparing it with a commercial formulation of fusidic acid. The Cur-NE showed the same results as the commercial formulation regarding wound contraction and re-epithelialization time. In terms of inflammation, Cur-NE significantly reduced inflammation (edema) in the carrageenan injection model. A bicontinuous ME containing *Melaleuca alternifolia* essential oil (MEO-loaded BME) was developed by de Assis and co-workers.^169^ The authors conducted an in vivo assay in Swiss mice (*Mus musculus*) over a period of 11 days. In vivo studies have demonstrated that the MEO-loaded BME effectively enhances the healing of skin wounds by increasing the percentage of wound edge contraction. Additionally, it exhibits antibacterial activity against both Gram-positive and Gram-negative bacteria. Moreover, the MEO-loaded BME at the highest concentration (3.45%) demonstrated a reduction in the inflammatory profile of the lesion, with a decrease of neutrophils and lymphocytes, as well as a lower angiogenic index.

Although there are many studies confirming the success of NE and ME formulations in the treatment of skin wounds through in vitro, in vivo, and even ex vivo tests, there is a gap when it comes to clinical trials. There are few articles that address the application of NE on skin wound human trials available in the main databases,^171−173^ and it is also notable how no articles focused on ME being applied in skin treatment clinical trials were found. Other works like those of Souto and co-workers^170^ and Chhabra and co-workers^154^ that also summarize different studies focused on the application of NE and ME in the skin have shown no articles that include a clinical trial. There are several barriers in starting a clinical trial, like financial bottlenecks, strict regulatory and ethical rules, lack of infrastructure, and difficulty in patient recruitment.^176^ Within all of the articles that did a human trial, the sample size was considered small. Still, even with all the challenges involved in its application, the presentation of a clinical trial is an essential step in affirming that a nanoformulation is suitable for approval as a pharmaceutical nanosystem and can subsequently be applied to the market.

Table 5 presents several studies in which NE were developed for wound treatment, along with the results obtained from clinical trials. As previously mentioned, no articles using ME on the subject were found.

**Table 5:** NE Development and Application on Skin Wound Treatments with Clinical Trial Results

type of nanoformulation	composition	model of trial	skin wound	outcomes	limitations	reference
NE-gel	decyl glucoside; water; hyaluronic acid; allantoin; aloe vera gel; d-panthenol; sodium benzoate; vitamin E; sea-buckthorn berry oil; carbomer; 5% r-r citric acid	sensory analysis and test of the effect of the nanoemulsion on the skin condition after burns	burned skin	improved hydration and elasticity of the skin smoothness	very small sample size (*n* = 4)	(171)
NE-hydrogel	guar gum; peppermint oil; menthol; methyl salicylate	multicenter, controlled trial	burn-induced hypertrophic scars	decrease in Pruritus severity and scratching the wound site; improvement in sleep quality, with the side effect of reported local irritations that were temporary and reversible	some patients left the study due to the local irritations caused by the formulation	(172)
atorvastatin-loaded NE	atorvastatin; sesame oil; polysorbate 80; propylene glycol; glycerol	randomized double-blind placebo-controlled clinical trial	postlaparotomy wound	decrease in redness, edema, ecchymosis, discharge, and approximation (REEDA scores)	the sample size was reduced due to COVID-19 pandemic; no statistic difference was found between the atorvastatin-loaded NE and an atorvastatin-loaded emulgel also developed and tested by the group	(173)

Regarding the patent deposition of these two nanosystems at the USPTO, it was possible to retrieve a total of 1,925 patents deposited from 1996 to 2024 using the combination of keywords described in the Methods for NE. In the period considered in this study (2012–2023), a total of 638 patents were found. Of this total, slightly over half (*n* = 334; 52.35%) were deposited in the last five years (2018–2023). Regarding ME, 4,072 patents were deposited in the period from 1991 to 2024 using the combination of keywords described in the Methods. In the period considered in the current study (2012–2023), a total of 864 patents were found. Of this total, slightly less than half (*n* = 417; 48.26%) were published in the last five years (2018–2023).

We emphasize that, unlike the research conducted for conventional formulations (cream, hydrogel, and ointment), there was no refinement stage in the data found at the USPTO for NE and ME due to the large number of elements generated in the search, thus extrapolating the actual number of patents deposited with a bias toward the production of a nanosystem for the treatment of topical wounds.

In any case, it is possible to observe that the deposition of patents for works that have developed these nanosystems is higher than that observed for conventional ones. This characteristic reflects the interest of researchers and industries. A brief survey in the Web of Science database conducted by Liu and co-workers^174^ identified that from January 1999 to December 2022 a total of 100,192 publications were found using the term “nanomedicine”. This interest is linked to the fact that nanosystems, in general, have several advantages over conventional drug delivery systems such as protection of the active ingredient against physical-chemical instabilities (e.g., hydrolysis, thermal instability, photosensitivity), dose reduction and consequently reduced toxicity and adverse effects, the possibility of prolonged release of the active ingredient, bypassing first-pass metabolism when administered orally, and targeting the nanosystem to the treatment target site (passive or active), among others.^174−177^

Notwithstanding, the emergence of nanotechnology does not take conventional systems (hydrogel, emulsion, and ointment) to ostracism.^178^ Their development is simple and usually safe for human health. In addition, regulatory agencies have expertise in the analyses of these products, which are extensively accepted by the market and consumers. Furthermore, despite nanosystems’ advantages, the development of nanoscale products is difficult and has a challenging scale-up process, often cost issues, and a bureaucratic and regulatory burdensome process to get consumers.^119^

Table 6 presents a comparative frame between the conventional pharmaceutical forms and nanobased emulsions for wound treatment.

**Table 6:** Comparison of Different Formulation Types for Drug Delivery and Performance Characteristics (Nano and Conventional Forms)^a^

characteristics	hydrogels	emulsions	ointments	NE/ME
delivery of both hydro and lipophilic substances	X	√	X	√
washability	√	X	X	√
wound protection against external threats	√	√	√	√
spreadability	√	+/–	+/–	√
reduction of dose of active ingredients	X	X	X	√
protect drugs from hydrolysis and oxidation	+/–	√	X	√
controlled drug release	+/–	+/–	X	√
intimate contact between the formulation and the cellular structures	X	X	X	√
expertise of regulatory agencies	√	√	√	X
solid regulatory framework	√	√	√	X
consumer acceptance	√	√	√	+/–

a NE = nanoemulsion; ME = microemulsion.

## 6 Challenges in Regulation of Pharmaceutical Nanosystems

Nanotechnology plays a crucial role in the technological and innovative development of companies and nations and has received increasing support through various government funding programs in recent years.^179^ The USA government invested approximately $40.7 billion between 2001 and 2023 through the National Nanotechnology Initiative. In 2020 alone, the Chinese government invested over $1.5 billion, while the European Union (EU), through the Horizon 2020 program, allocated more than €2.7 billion between 2014 and 2020.^180,181^

Alongside scientific, technological, and strategic interests, regulatory concerns have emerged, driven by ethical, legal, human health, and environmental considerations.^182^ Globally, various initiatives have been undertaken to discuss the regulation of nanomaterials. However, despite these efforts, there are still no established regulations for registering nanomedicines—only guidelines.^183^ For instance, the U.S. Food and Drug Administration (FDA) published *Drug Products, Including Biological Products, that Contain Nanomaterials – Guidance for Industry* (FDA-2017-D-0759).^184^ Meanwhile, the European Medicines Agency (EMA) has issued several guidelines, including *Data Requirements for Intravenous Liposomal Products Developed with Reference to an Innovator Liposomal Product – Scientific Guideline* (EMA/CHMP/806058/2009/Rev. 2)^185^ and *Surface Coatings: General Issues for Consideration Regarding Parenteral Administration of Coated Nanomedicine Products* (EMA/325027/2013).^186^ These documents provide scientific and regulatory guidance but do not yet constitute formal regulatory frameworks for nanomedicine registration.

A regulatory framework for nanomedicine faces many challenges. Nanosized materials exhibit novel biophysical and chemical characteristics that, when in contact with the human body, can lead to toxic effects not observed in micrometric materials, such as enhanced permeation across the blood–brain barrier and increased deposition in the lungs and liver.^187^ The pharmacokinetics of nanomaterials presents another challenge for regulatory agencies, as nanomedicines may have sustained release profiles and deviate from the expected metabolism of drugs in conventional pharmaceutical forms.^188^ Other challenges such as physicochemical characterization, process control, scale-up, and reproducibility are bottlenecks in nanosized materials and nanomedicine development and regulation.^188^ The global classification of nanomaterials and nanomedicines is not standardized, which can complicate their registration in different countries.^189^ Therefore, a coordinated international effort is needed to harmonize definitions, classifications (e.g., as medical devices or nanomedicines), and required assays to ensure nanosafety, once the nanotechnology is in rapid development.^190^ This is currently being addressed through the International Pharmaceutical Regulators Programme Nanomedicine Working Group.^191^

Regarding NE and ME, some nanomedicines are approved and available at market, such Sandimmun Neoral, Restasis (Cyclosporine), Oncaspar (Pegaspargase), Fortovase (Saquinavir), Norvir (Ritonavir), Propofol (Propofol), Vitalipid (vitamins A, D, E, and K), and Durezol (Difluprednate).^192^ Despite that, none of these nanomedicines have clinical indications for wound treatment, and no NE or ME was identified for this application in our research even in the market or at the clinical trial stage at ClinicalTrials.gov or ClinicalTrialsRegister.eu, but a number of articles can be found in an easy way at databases proposing NE and ME for wound treatment. These nanosystems have been used so far for dermal applications such as cosmetics because of their advantages such their stability, large surface area, and sustained release of bioactive ingredients.^193^ Beyond their technological advantages, nanocosmetics have a clearly regulatory framework compared to nanomedicines, such in EU with European Commission Regulation No. 1223/2009, which establishes standards for cosmetic products, ensuring consumer safety and the proper functioning of the internal market.^194^

## 7 Conclusions

Wounds can be installed in a periodic process due to imbalances between the components of the web of the healing process, affecting the life of the individual with the injury as well as resulting in high expenses for governments and companies for individuals with chronic injuries. Using classic systems for delivering active substances, such as emulsions, gels, and ointments, to the injury site is extremely important to alleviate the above-mentioned issues. In the last 10 years, it has been possible to observe a low number of patents filed using these systems; however, this fact has not diminished the historical importance and technological potential of these conventional systems. Nanotechnology is an excellent tool for delivering drugs for the treatment of various diseases including wound management. The interest of researchers and industry in nanosystems, especially NE and ME, can be evidenced by the high number of patents filed in the period analyzed. In the future, it is expected that there will be an increasing number of publications on the use of nanosystems; however, conventional dosage forms continue to be responsible for a large share of the consumer market due to broad public acceptance. The lack of NE and ME applications on skin wound studies including clinical trials, considering the wide results with in vitro and in vivo tests, shows that there is a new barrier to be overcome so that these nanoformulations can be introduced into the pharmaceutical market.

## 8 Methods

For topics related to skin anatomy and physiology, the combination “skin” AND “structure” was used; for articles related to healing, the term “wound healing process” was used using Web of Science, ScienceDirect, and PubMed as databases. Regarding the topic of conventional formulations used to treat wounds, Google Scholar was used, with the search using the following combinations: “hydrogel” AND “review”, “emulsion” AND “review”, and “ointment” AND “review”. To search for patents filed in 2012–2023, the United States Patent and Trademark Office database was selected using the following combinations: “hydrogel” AND “topical wound treatment”, “emulsion” AND “topical wound treatment”, “ointment” AND “topical wound treatment”, “nanoemulsion” AND “wound” AND “topical” AND “treatment”, and “microemulsion” AND “wound” AND “topical” AND “treatment”. To search for clinical trials using nanoemulsion or microemulsion on the treatment of skin wounds, the same databases were used with the combination “microemulsions” OR “nanoemulsions” AND “skin wound”, excluding articles that had not performed a clinical trial.

## Abbreviations

EGF: epidermal growth factor
EMA: European Medicines Agency
Ep: epidermis
EU: European Union
FDA: U.S. Food and Drug Administration
FGF-2: fibroblast growth factor-2
HLB: hydrophilic–lipophilic balance
Ke: keratin
ME: microemulsion
NE: nanoemulsion
PaD: papillary dermis
PAF: platelet-activating factor
PDGF: platelet-derived growth factor
ReD: reticular dermis
ROS: reactive oxygen species
TGF-α: transforming growth factor-α
TGF-β: transforming growth factor-β
TNF-α: tumor necrosis factor-α
USA: United States of America
USPTO: United States Patent and Trademark Office
VEFF: vascular endothelial growth factor
